# Changes in R0/R∞ ratio and membrane capacitance are associated with milk removal from the breast

**DOI:** 10.1371/journal.pone.0208650

**Published:** 2018-12-07

**Authors:** Hazel Gardner, Ching Tat Lai, Leigh Ward, Donna Geddes

**Affiliations:** 1 School of Molecular Sciences, Faculty of Science, University of Western Australia, Perth, Australia; 2 School of Chemistry and Molecular Sciences, University of Queensland, Brisbane, Queensland, Australia; University of Illinois, UNITED STATES

## Abstract

Perceived low milk supply is a common reason for introducing supplementary feeds, which in turn serves to further diminish the milk supply. Current methods of measuring milk production and milk transfer from the breast to the infant are inaccessible to the mothers. There is a need for an inexpensive, portable device to enable mothers to measure milk transfer to either confirm their milk production is adequate or identify breastfeeding issues early. The aim of this study was to examine changes in bioimpedance spectroscopy associated with milk removal from the human lactating breast using an electric breast pump. Thirty lactating women participated in 2 research sessions performed in random order over 2 weeks. Milk flow rate and volume were measured during pumping. All mothers completed 24-hour milk profiles. Breasts were monitored using bioimpedance spectroscopy. Analysis was performed using linear mixed effects models to investigate the relationship between both proportional change in membrane capacitance (C_m_) and R0/R∞ with milk removal. There was an inverse relationship between R0/R∞ and milk removed (p<0.001). A positive relationship was also observed between C_m_ and both volume of milk removed (P<0.001) and percentage of available milk removed (p<0.001). This study has shown that changes in bioimpedance are related to the volume of milk removed from the breast during pumping. This modality may hold promise for the measurement of the effectiveness of the breastfeeding infant in removing milk from the breast.

## Introduction

One of the most common concerns for first time mothers is the adequacy of their milk supply in meeting the needs of the infant. Perceived low milk supply (PLMS) is the most common reason for introducing supplementary feeds, which in turn serve to further diminish the milk supply [1]. Maternal stress associated with PLMS may also potentially have a negative impact on milk transfer, affecting both milk ejection and ultimately milk synthesis [2].

In order to provide sufficient breast milk, milk must be synthesised by the lactocytes and then, through milk ejection, be propelled towards the nipple for removal by the infant or breast pump. For breastfeeding to be successful the infant must actively remove milk through the application of a cyclic vacuum [3]. The strength of the vacuum and the volume of milk in the breast are determinants of how well the breast is drained. Frequent emptying of the breast is associated with increased rates of milk synthesis [4–6]. Once establishment of milk production has occurred maintenance is under autocrine control, and therefore effective emptying of the breast by the infant is critical to successful lactation [7]. Risk of low milk supply is observed when the infant applies a weak vacuum, such as in the case of the breastfeeding preterm infant [8].

Infant growth is often used as a marker of adequate milk supply. However infant behaviour may also be indicative of the infant not receiving adequate volumes. In this case, it is important for mothers to ascertain whether any insufficiency in breast milk volume is real or perceived, as low milk supply may have a detrimental impact on the optimal growth and development of the infant, and therefore supplementation is indicated [1, 9]. Conversely, if the milk supply is adequate and the infant’s requirements are being met, unnecessary supplementation may itself cause a reduction in milk supply and hence shorten the duration of breastfeeding [10].

Previous methods of measuring milk removal from the breast have included measuring breast volume changes through the use of topography [11], a computerized breast measurement system [12], and test weighing of the mother or infant [13]. The use of breast volume to measure milk synthesis and milk removal proved to be impractical due to the amount of equipment and the complex data analysis required. However, test weighing [13] is increasingly being used in a clinical setting by health professionals to assess the intake of the infant over a twenty-four hour period [14]. This method is utilised by relatively few breastfeeding dyads, hence the need for an inexpensive, portable tool to enable mothers to measure their milk production.

Bioimpedance analysis, most commonly used for body composition assessment[15], has been used to measure the functional state of biological tissues[16], and is minimally invasive and easy to use. Bioimpedance has also been used in the characterisation of healthy breast tissue and breast pathologies [17–19]. This method involves passing a small current through bodily tissues, which is primarily conducted by the electrolytes in body fluids. Fluid is compartmentalised into intra- or extracellular spaces, each having different electrical conductivities but similar osmolarity. Cell membranes act as imperfect capacitors; i.e. conducting through the extracellular fluid only at lower frequencies, with the capacitive effect of the cell membrane decreasing at higher frequencies allowing the current to pass through both the intra- and extracellular fluids [20]. Breast milk is an extracellular fluid and thus bioimpedance measurements at low frequencies reflect the resistance as the current flows through the extracellular space (R0). At low frequencies, resistance is lower when levels of extracellular fluid are high. R infinity is an indicator of total fluid and therefore the R0/R∞ indicates changes in extracellular fluid in relation to total fluid. During milk removal from the breast there are large shifts in milk volume as milk is ejected from the alveoli into the ducts to be removed by the infant. It is hypothesised that this will be reflected by changes in low frequency resistance.

The aim of this study was to determine if there is a relationship between measures of bioimpedance of the breast and the volume of milk removed during milk expression.

## Materials and methods

Thirty lactating women were recruited through the Australian Breastfeeding Association or through social media. The study was approved by The University of Western Australia Human Research Ethics Committee (RA/4/1/7897) and all the participants provided written informed consent. All participants completed a background questionnaire to collect demographic information. Most of the research sessions were undertaken in participants’ homes with one participant completing all sessions in the laboratory at The University of Western Australia.

### Milk production

Each participant was issued with a set of baby weigh scales (Medela AG, Switzerland) and completed a 24 hour milk profile. This involved recording all breastfeeds and milk expressed during the 24 hour period, using the test weighing method [13]. Milk intake may be underestimated by 10 ±12% as no correction was made for infant insensible water. Infants were weighed and milk samples of 1–2 ml were collected in 5 ml polypropylene tubes (P5016SL, Techno Plas Pty Ltd, SA, Australia) before and after each feed. Five participants expressed milk during the 24 hour period and they collected samples pre- and post- breast expression and milk volume was measured. The expressed milk was either fed to the infant during the 24 hours or was stored for future use. All of the data was entered by the participants, along with the time, to an app. Milk samples were frozen until they could be collected and transported to the laboratory, where they were analysed for fat content using the creamatocrit method [21]. The electronic data entered by the participants was then used to calculate breast fullness and storage capacity, based on the method described by Kent et al, [22]. The volume and cream content of milk removed during the experimental sessions were added to this dataset to facilitate calculation of available milk, breast fullness and percent available milk removed (PAMR) during each session.

### Milk flow

**Fig 1:**
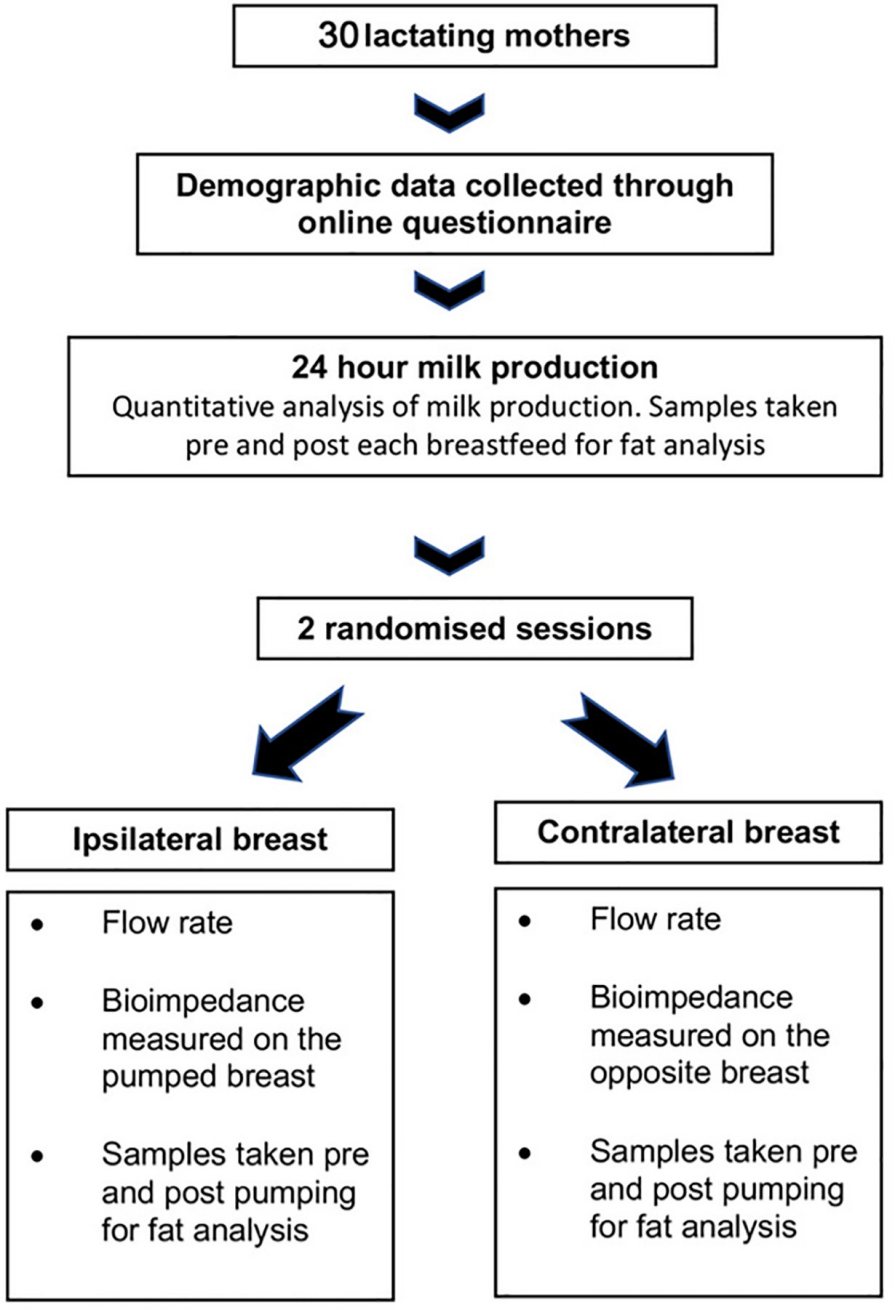
Study design.

Data collection sessions were conducted a week apart (Fig 1) at the same time of day using a Symphony hospital-grade pump (Medela AG, Baar, Switzerland). The time since last feed or expression differed between participants and between sessions. However this was accounted for through the calculation of available milk, breast fullness and percent available milk removed. Milk flow rate during the expression of milk with an electric breast pump was measured using the method described by Prime et al [23] using a continuous weighing balance to measure cumulative milk and flow rate in grams per second [23]. The balance had a resolution of 0.1g and accuracy of ± 0.02% and was connected to a computer through a USB port. Cumulative milk volume data were sampled at 8 Hz and a derivative was used to calculate flow rate in grams/second (Showmilk, Medela AG, Switzerland).

### Bioimpedance

**Fig 2:**
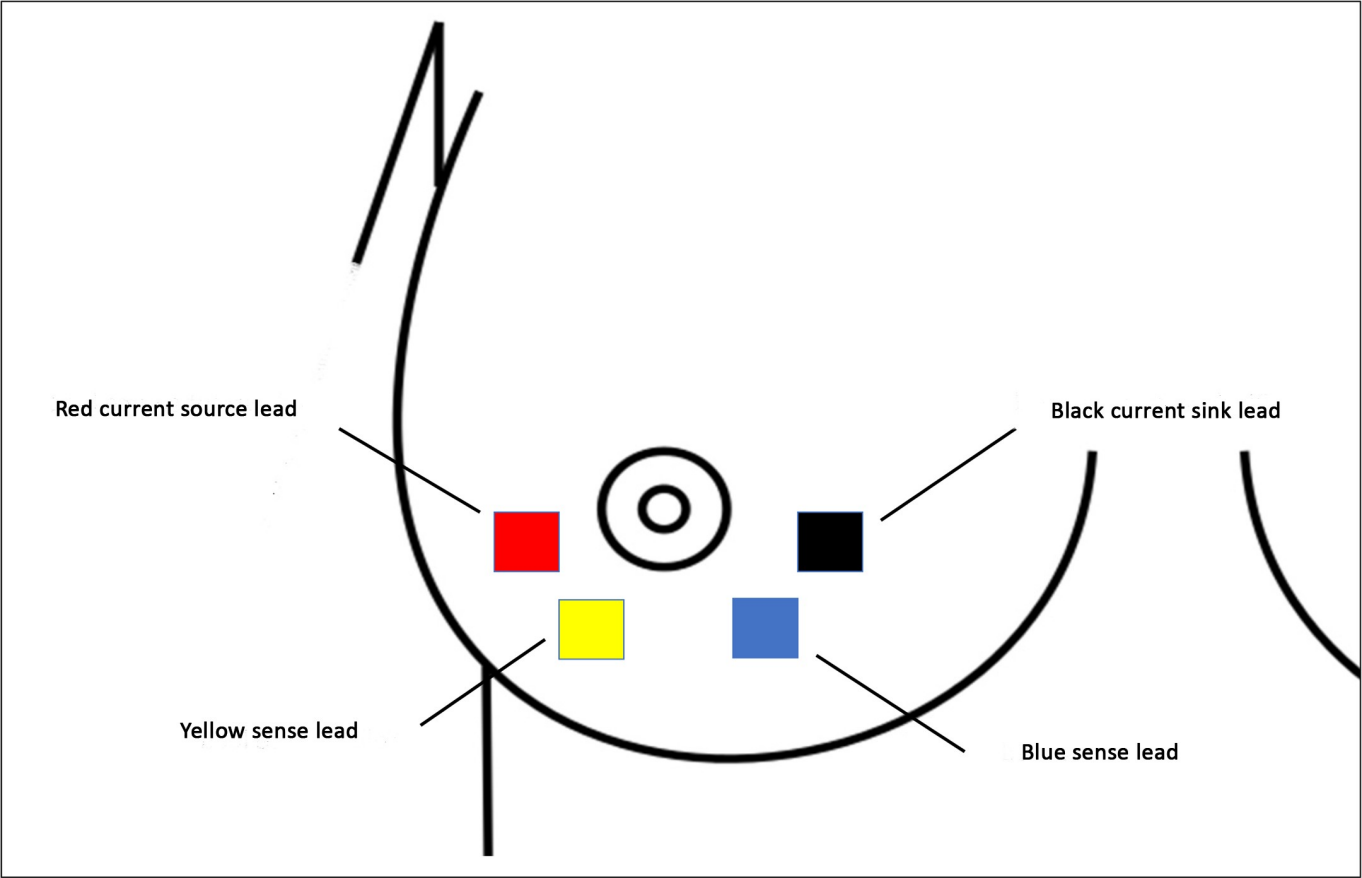
Diagrammatic representation of electrode placement.

Bioimpedance data were collected using an Impedimed SFB7 bioimpedance spectroscopy machine, which measures bioimpedance parameters (impedance and it’s components, resistance and reactance) at 256 frequencies between 3–1000 kHz. The SFB7 is a tetrapolar device, using separate drive current and impedance sensing circuits. A harmless current (200 μA) is applied via distally located electrodes, with sense electrodes spanning the measurement region. The breast was swabbed with an alcohol wipe. EKG-style gel pad electrodes (Impedimed Pinkenba, QLD 4008, Australia) were placed on the inferior medial and inferior lateral quadrants of the breast (Fig 2) such that sense electrodes encompassed the majority of the mammary secretory tissue. The experimental breast was randomly selected during the first research session, and the electrodes were placed in the same positions on the same breast for the duration of the study. There was a two-minute calibration period, before the pump was switched on, to collect baseline data. Bioimpedance data were collected continuously for the duration of each session.

Each participant took part in two research sessions (Fig 1). The duration of the pumping sessions was 10 minutes post-milk ejection; one session with the electrodes on the breast being pumped (ipsilateral) and the other on the contralateral breast. The milk flow data and the bioimpedance data were manually synchronised for each session in preparation for analysis.

### Statistical analysis

Bioimpedance data were fitted to Cole plots (reactance versus resistance) and analysed using the Bioimp (version 5.4.03) software provided with the SFB7 machine (Impedimed Pinkenba, QLD 4008, Australia). Resistance values at zero and infinite frequencies (R0 and R∞ respectively) and membrane capacitance (C_m_) were determined for each frequency scan and plotted against time over the pumping session. The ratio between R0 and R∞ was calculated. Membrane capacitance measures were taken at the beginning and end of each session and the changes were converted into a proportion of the starting value. Linear mixed effect models were used to analyse pumped milk volume, PAMR, volume of milk removed over time, R0/R∞ infinity ratio and change in C_m._ The volume of milk removed and PAMR were modelled with the fixed effect of pump session. The volume of milk removed over time was modelled with fixed effect of either the R0/R∞ infinity ratio or change in C_m._ PAMR was modelled with fixed effect of change in C_m._ All models considered random effects for the participant. Linear regression was used to illustrate the correlation between changes in C_m,_ milk volume removed and PAMR. Statistical analyses were carried out with RStudio Version 1.0.136 [24] using package nlme [25] for linear mixed effect modelling. Data are presented as mean ± standard deviation (SD). Significance was set at the 5% level.

## Results

Participant characteristics are shown in Table 1. The participants came from a broad geographical area in the greater Perth region. The lactating women who took part in the study had a wide range of body mass indices, parities and stages of lactation.

**Table 1:** Maternal and infant characteristics in 30 mother-infant dyads.

		Median	Range
**Mother**	Age (years)	32	24–38
	Parity	1	1–5
	BMI (kgm^-2^)	26	19–54
**Infant**	Gestational age at birth (weeks)	39	37–41
	Birthweight (g)	3642	2660–4645
	Current age (weeks)	17	5–33
**Milk** **production**	Storage capacity left breast (ml)	168	84–455
	Storage capacity right breast (ml)	184	88–286
	Milk produced from left breast (ml)	363	228–741
	Milk produced from right breast (ml)	380	221–723
	Total volume (ml) milk production in 24-h (breastfeeds and expressions)	773	484–1155

Milk removal characteristics from the pumped breast are detailed in Table 2. Contralateral breast refers to the electrode placement on the opposite breast. The milk volume pumped and PAMR (percent available milk removed) were similar between sessions (Table 2) (volume: p = 0.17; PAMR: p = 0.61).

**Table 2:** Milk removal characteristics from the two monitored pumping sessions.

Measure	Ipsilateral breast (mean ± SD)	Contralateral breast (mean ± SD)
Breast fullness	0.7 ± 0.2	0.7 ± 0.3
Available milk (ml)	144 ± 64	136 ± 65
Milk volume (ml)	81 ± 55	70 ± 51
% Available milk removed	58 ± 26	55 ± 27
Number of milk ejections	3 ± 1	3 ± 1

**Fig 3:**
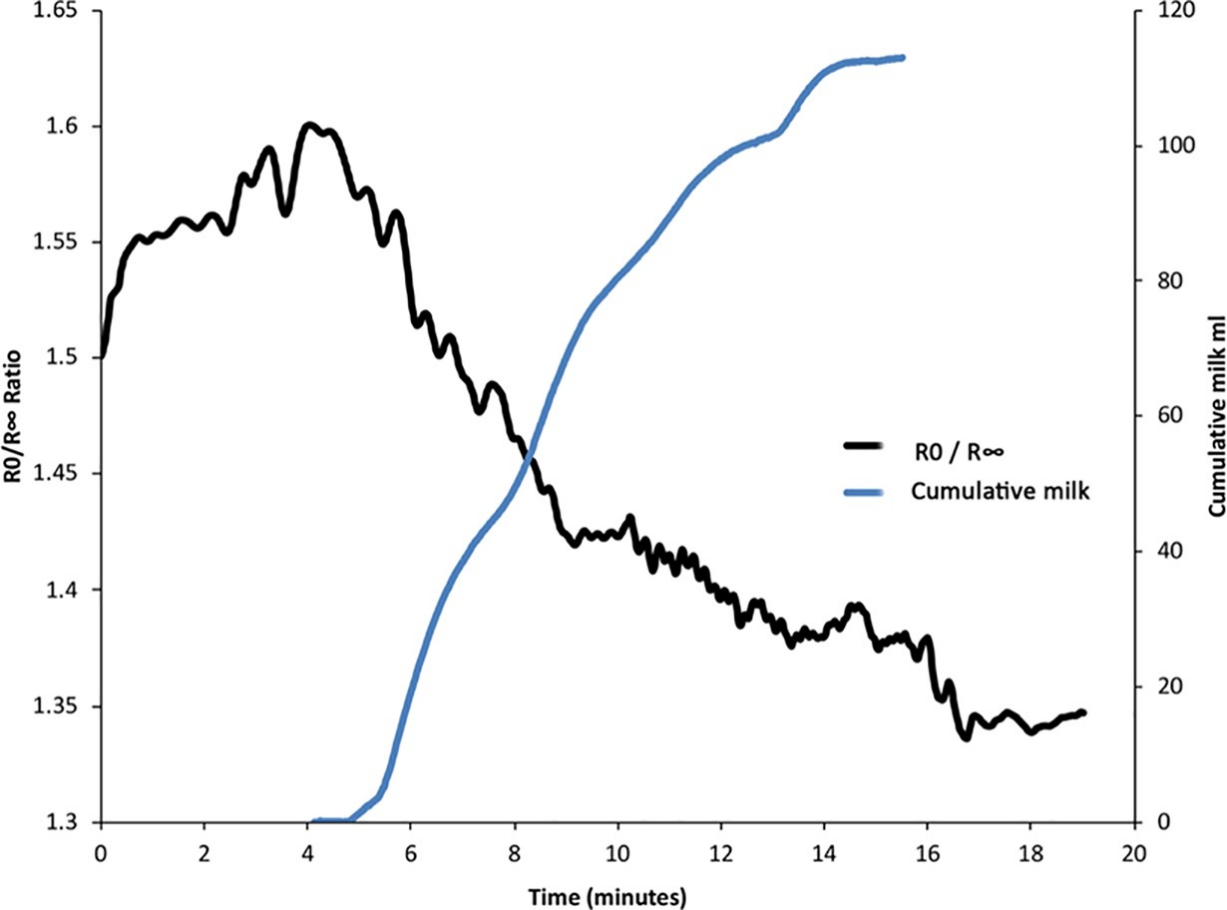
The inverse relationship between the R0 /R ∞ ratio and cumulative milk removed for a typical pumping session.

The R0/R∞ ratios were plotted for each session and overlaid with synchronised cumulative milk output (Fig 3). Analysis of these plots indicated a negative relationship between the R0/R∞ infinity ratio and the volume of milk removed (p<0.001, R^2^ = 0.23) over time when the ipsilateral breast was pumped. This relationship was not found when electrodes were placed on the contralateral breast, where milk was not removed.

**Fig 4:**
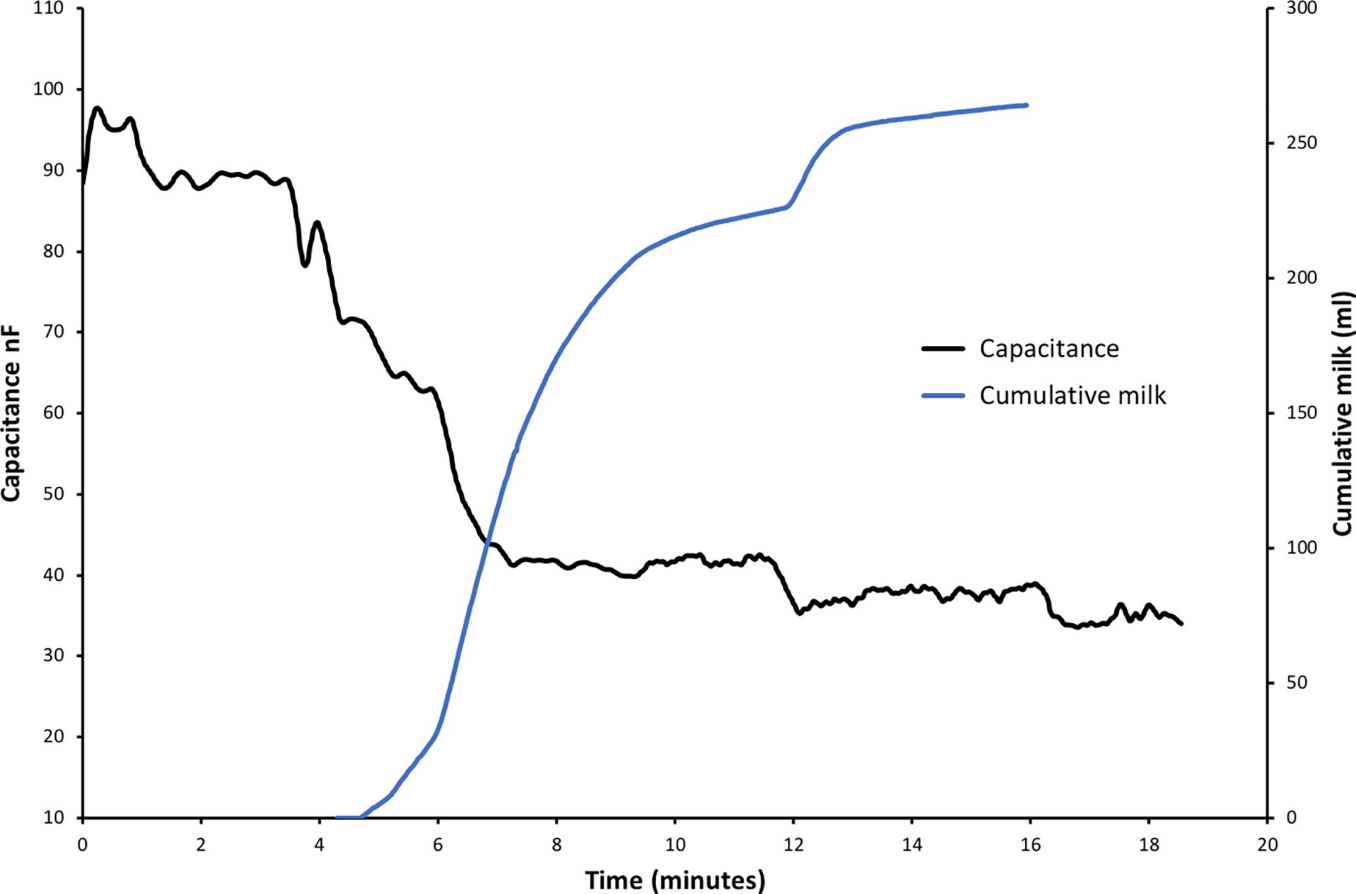
The inverse relationship between membrane capacitance and cumulative milk removed for a typical pumping session.

Membrane capacitance also decreased and the mean proportional change was 0.4 ± 0.2 (0.04–0.7). This decrease in C_m_ occurred as the volume of milk increased (Fig 4.).

**Fig 5:**
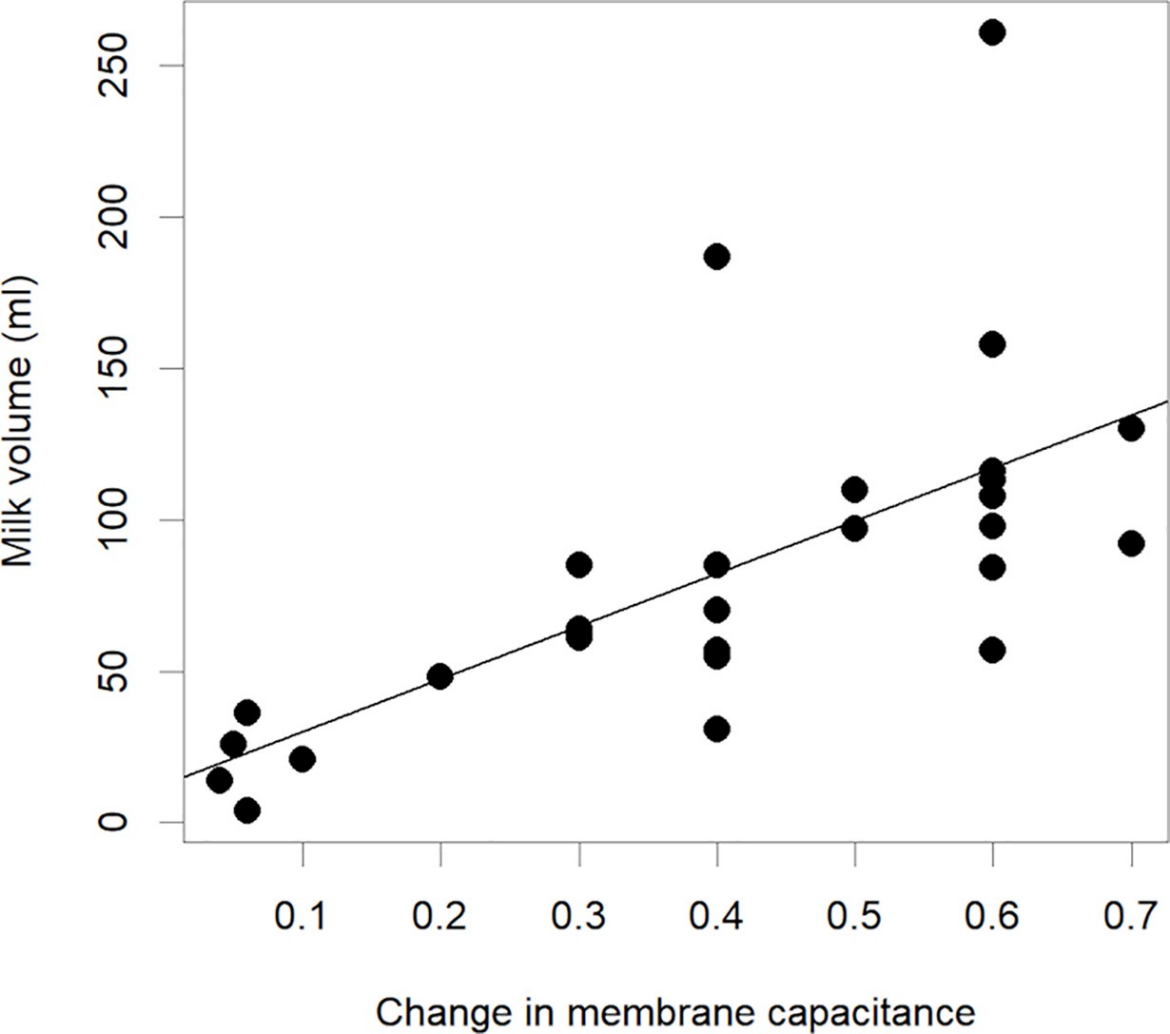
The relationship between the proportionate change in membrane capacitance of the ipsilateral breast and milk volume removed from the breast for all subjects.

Utilising the proportionate change in C_m_ as a variable, a significant correlation was found between the change in C_m_ and the volume of milk removed in the ipsilateral breast over the entire pumping session (P<0.001, R^2 =^ 0.42) (Fig 5.).

**Fig 6:**
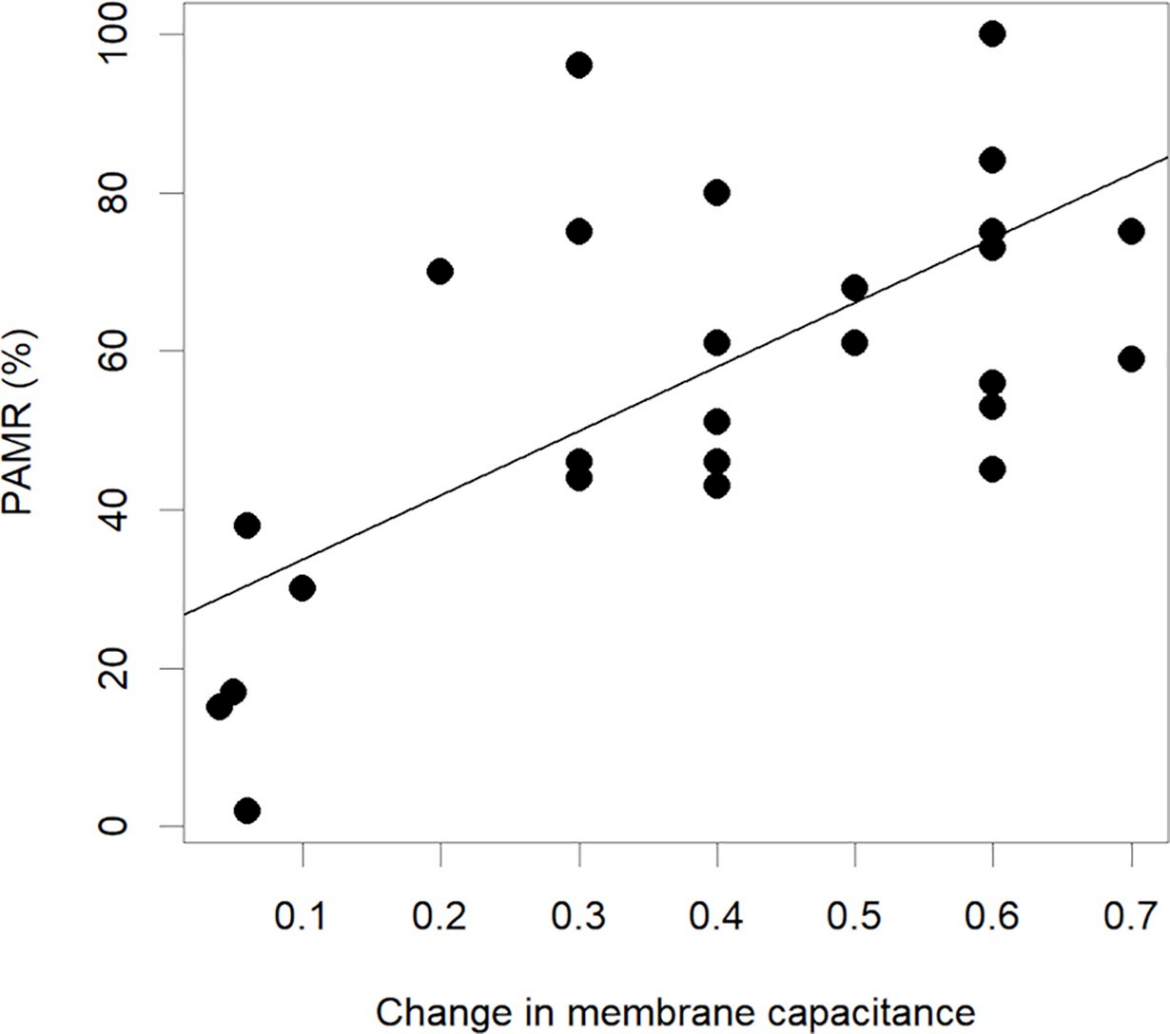
The relationship between the proportionate change in membrane capacitance and percentage available milk removed (PAMR) from the breast for all subjects.

PAMR was also significantly related to the change in C_m_ when measured on the pumped breast (P<0.001, R^2^ = 0.41) (Fig 6.). This relationship was not observed when electrodes were placed on the contralateral breast during pumping.

## Discussion

The results from this study confirm that bioimpedance characteristics are related to milk removal from the breast during breast expression with an electric breast pump. We found that R0/R∞ and C_m_ were related to milk volume removed and PAMR.

Monitoring of the lactating breast with bioimpedance during pumping illustrates that as the R0/ R∞ ratio decreases, the volume of milk removed increases. The ratio decreases, indicating a rise in extracellular fluid levels in the monitored area of the breast. Milk is an extracellular fluid secreted by lactocytes lining the mammary alveoli. At low frequencies, the current applied through the bioimpedance device cannot penetrate the cells and only the extracellular fluid is measured. Impedance ratios are commonly used in physiological research [26, 27] such as in dialysis [28], and are considered to provide information on fluid redistribution and data reliability. At milk ejection, the ducts expand during the period of milk removal, as they transport milk towards the nipple [29]. As milk is removed from the ducts, their diameter will decrease. The reduced volume of milk within the ducts will then result in a reduction in the ratio. Another potential contribution to the mammary extracellular fluid is the accumulation of interstitial fluid, which is drawn into the area as a result of the negative pressure applied by the pump, and is later cleared through the lymph system [30]. To confirm this speculation, extended monitoring of the breast post-pumping, may show a potential increase in the ratio as fluid is dissipated [31].

In conjunction with the observed decrease in R0/R∞ ratio larger decreases in C_m_ were related to both greater volumes of milk removed from the breast and higher PAMR. C_m_ is associated with membrane surface area of cells, and therefore increases with increased hydration, and is also impacted by normal cell functioning of ion channels and pumps [32]. The changes observed in C_m_ during milk removal could be related to the changes in both physical structure and physiology of the breast. For example blood flow increases at the first milk ejection and then decreases across a feed. A reduction in mammary blood may partially account for the decrease in MC across time. Further, the removal of milk volume itself would likely contribute to a reduction in C_m_ since, as noted above, milk removal involves changes in duct diameter which implies a change in lining cell membranes which may also contribute to the observed change in C_m._ Other potential variables include changes in breast shape and pressure. Further research is necessary to elucidate the mechanism behind the changes in mammary C_m_.

While the relationships between the R0/R∞ ratio and C_m_ with milk removed were not found to be predictive of the volumes removed, a larger sample size and multiple data collection sessions with the same individuals may improve the predictive power. Furthermore, testing is required during breastfeeding to determine if the relationships found during pumping are also prevalent during feeding.

Determination of the optimal site for electrode placement meant the milk removal from the lobes in the lower portion of the breast was monitored. The lobes are arranged in such a way that most of the ductal activity occurs in the inferior and lower lateral quadrants of the breast [33] despite the increase in glandular tissue volume during lactation [34]. We were able to obtain robust measurements in this region due to the high synthetic and ejection activity in the lower half of the breast compared to other areas.

Whilst this is a relatively small sample size, the study participants had a wide range of BMI, parities and stages of lactation, which suggests that bioimpedance could potentially measure milk removal in mothers with a wide range of characteristics. Further, the women exhibited variable levels of milk production, potentially allowing the detection of changes in the breast when milk supply is not optimal. To confirm these results future studies would include larger numbers of women in each stage of lactation as well those with low milk supply.

The limitations of the study are that the bioimpedance device is primarily designed for whole body composition analysis. Optimisation of electrode design and software for breast measurement for the present novel application may prove advantageous [35]. Due to the anatomy and shape of the breast, we were limited to a small area of the breast, and therefore the results may not be representative of the entire breast.

## Conclusion

This study has shown that, when the lactating breast is monitored with bioimpedance during pumping, the volume of milk removed and per cent available milk removed are strongly associated with decreases in both the R0/R∞ ratio and C_m_. Optimisation of this method may provide a non-invasive tool to monitor milk removal from the breast during breastfeeding.

## Supporting information

S1 Data Raw data for publication. (XLSX)
